# A new record of *Ganodermatropicum* (Basidiomycota, Polyporales) for Thailand and first assessment of optimum conditions for mycelia production

**DOI:** 10.3897/mycokeys.51.33513

**Published:** 2019-05-07

**Authors:** Thatsanee Luangharn, Samantha C. Karunarathna, Peter E. Mortimer, Kevin D. Hyde, Naritsada Thongklang, Jianchu Xu

**Affiliations:** 1 Key Laboratory for Plant Diversity and Biogeography of East Asia, Kunming Institute of Botany, Chinese Academy of Sciences, Kunming 650201, Yunnan, China; 2 University of Chinese Academy of Sciences, Beijing 100049, China; 3 East and Central Asia Regional Office, World Agroforestry Centre (ICRAF), Kunming 650201, Yunnan, China; 4 Centre for Mountain Ecosystem Studies (CMES), Kunming Institute of Botany, Kunming 650201, Yunnan, China; 5 Center of Excellence in Fungal Research, Mae Fah Luang University, Chiang Rai 57100, Thailand

**Keywords:** Cultivation, medicinal mushroom, morphological characteristics, phylogeny, taxonomy

## Abstract

In this study a new record of *Ganodermatropicum* is described as from Chiang Rai Province, Thailand. The fruiting body was collected on the base of a living *Dipterocarpus* tree. The sample is described on the basis of morphological characteristics and phylogenetic analyses, and compared with closely related taxa. Multigene phylogenetic analyses of LSU, ITS, and RPB2 highly support the placement of the *G.tropicum* group with isolates from China and Taiwan (Maximum likelihood 100%, Maximum parsimony 100%, and Bayesian posterior probabilities 1.00). The optimal media, pH, and temperature for mycelial growth of the *G.tropicum* strain KUMCC18-0046 was also investigated and is reported as: PDA, MEA, and YPD, at pH 7–8 and 25–28 °C, respectively. This is the first report on the successful growing conditions for mycelial production, but unfortunately fruiting could not be achieved.

## Introduction

*Ganoderma* P. Karst. was established as a white rot fungus (Ryvarden 2004), showing parasitic or pathogenic behavior on wide range of tree species (Ryvarden 2004; Pilotti 2005; Dai et al. 2007). *Ganoderma* is more frequently distributed in tropical and temperate regions worldwide (Cao and Yuan 2013), and the distribution of *G.tropicum* is limited to the tropics (Cao et al. 2012). *Ganoderma* is characterized by distinctive laccate or non-laccate, sessile to stipitate basidiomata, double-walled basidiospores, and interwall pillars (Karsten 1881; Moncalvo and Ryvarden 1997). *Polyporuslucidus* (Curtis) Fr. is the original type species of the genus (Moncalvo and Ryvarden 1997). There are 449 records in the Index Fungorum (http://www.indexfungorum.org/; accessed date: 25 January 2019) and 384 records of taxa in MycoBank (http://www.mycobank.org/; accessed date: 25 January 2019).

*Ganoderma* produces a high number of natural bioactive compounds, such as polysaccharides, triterpenoids, sterols, and secondary metabolites (i.e. ganoderic acid, ganodermanondiol, ganodermanontriol, and ganodermadiol), which can be used to remedy a wide range of diseases (Richter et al. 2015; Hapuarachchi et al. 2018b). Many compounds have been found in different species of *Ganoderma*, and extracts derived from *G.tropicum* contain phenolic compounds such gano-dermatropins A (1) and B (2), as well as compounds with antimicrobial activity (Hu et al. 2013). *Ganodermatropicum* is recognized as a medicinal mushroom and has been recorded in the Chinese Pharmacopeia (Wu et al. 2013). The fruiting bodies contain natural triterpenes, primarily lanostanoid-type triterpenes, with potential use in chemotaxonomy (Ríos et al. 2012; Da Silva et al. 2013; Zhang et al. 2015).

The taxonomy of *Ganoderma* has been a constant topic of debate due to the high levels of phenotypic plasticity in species such as *G.lingzhi*, *G.lucidum*, and *G.sichuanense* (Pilotti et al. 2004; Wang et al. 2009; Cao et al. 2012; Dai et al. 2017; Loyd et al. 2018; Hapuarachchi et al. 2019). In an attempt to further our knowledge of the taxonomy this genus, we describe a specimen of *Ganodermatropicum* as a new record for Thailand based on morphological characteristics and phylogenetic analyses, and the optimal conditions for mycelial growth of *G.tropicum* are also reported.

## Methods

### Sample collection and isolation

In October 2017, a single fresh basidiocarp of *Ganodermatropicum* was collected on a living *Dipterocarpus* tree in a deciduous mixed rainforest dominated by *Castanopsis* and *Dendrocalamusstrictus* during the dry season. The coordinates of the described area in Chiang Rai Province, Thailand are 19°49'23"N; 100°01'41"E, 41 m. The sample was then photographed and transported back to the laboratory where its fresh macroscopic details were described. The culture was aseptically isolated by using heat sterilized forceps, transferring sections of internal tissue from fruiting bodies onto potato dextrose agar (PDA) and incubated at 25 °C, for 21 days, under dark conditions (Luangharn et al. 2017). After incubation, the agar surface was fully covered with white mycelium. The pure stock culture was then covered with mineral oil and deposited in the voucher culture collection of the Kunming Institute of Botany culture collection under the accession number KUMCC18-0046. The cultures are being maintained at 4 °C for further studies. The sample was then air dried at 45 °C for 48 hours until it was completely dehydrated. Finally, the herbarium material was deposited in the Herbarium of Mae Fah Luang University, Chiang Rai, Thailand (voucher number MFLU Herb. 17-1934) with duplicates in the Herbarium of Kunming Institute of Botany, Academia Sinica (HKAS), Yunnan Province, China (voucher number HKAS 97486).

### Morphological study

Macro-morphological characteristics were described following the method by Lodge et al. (2004), while colors were recorded following (Ridgeway 1912). Macroscopic characteristics were determined according to the methodology described by Largent (1986). To observe microscopic characteristics, free-hand sections were made under a dissecting microscope (OLYMPUS SZ61) and mounted on a glass slide in 3–5% KOH, 1–3% Congo red, and Melzer’s reagent for highlighting all tissues (Kreisel and Schauer 1987). Microphotography was done with a Nikon ECLIPSE Ni (Nikon, Tokyo, Japan) compound microscope, with a Canon EOS 600D (Tokyo, Japan) digital camera fitted on the top of the microscope. Basidiospores and hyphal system sizes, colour, and shapes were recorded and photographed. Measurements were taken using the Tarosoft® Image Framework program v. 0.9.0.7. The size and shape of basidiospores were followed [*Q = L/W*] and calculated considering the mean value of the lengths and widths in side view. The calculation was done by using at least 50 basidiospores from each basidiomata (Miettinen and Larsson 2006). The photographs were edited in Adobe Illustrator CS v. 3.

### DNA extraction, PCR amplification, and sequencing

Dried internal tissues of the basidiocarp were ground and total DNA was extracted using the Biospin Fungus Genomic DNA Extraction Kit (BioFlux). The ITS, LSU, and RPB2 genes were amplified by Polymerase Chain Reaction (PCR). The PCR amplifications were performed in a total volume of 25 μL of PCR mixtures containing 9.5 μL ddH_2_O, 12.5 μL of PCR master mix, 1 μL of DNA template, and 1 μL of each primer (10 μM). PCR amplification was carried out using primer pairs LROR/LR5 for the nuclear ribosomal large subunit 28S rDNA gene (LSU), ITS5/ITS4 for internal transcribed spacer rDNA region (ITS1, 5.8S rDNA and ITS2), and fRPB2-5F/fRPB2-7cR for the partial RNA polymerase second largest subunit region (RPB2) (Vilgalys and Hester 1990; White et al. 1990; Liu et al. 1999). The PCR cycling amplification conditions incorporated the following modifications: LSU initial denaturation was at 95 °C for 3 min, followed by 35 cycles at 95 °C for 30 s, 56 °C for 45 s, 72 °C for 1 min, and a final extension of 72 °C for 10 min. The PCR cycling for ITS was as follows: initial denaturation at 95 °C for 3 min, followed by 35 cycles at 95 °C for 30 s, 55 °C for 1 min and 72 °C for 1 min and a final extension of 72 °C for 10 min. The PCR cycling for RPB2 was as follows: initial denaturation at 96 °C for 3 min, followed by 35 cycles at 95 °C for 35 s, 54 °C for 10 s, 72 °C for 10 s, and a final extension of 72 °C for 5 min. The sequencing of PCR products was carried out by Sangon Biotech Co., Shanghai, China. The nuclear ribosomal Internal Transcribed Spacer region (nrITS) of the fungi was amplified and the sequence was deposited in GenBank to obtain the accession number.

### Sequence alignment and phylogenetic analyses

The sequence of the new record was subjected to standard BLAST searches of GenBank to determine the primary identity of the fungal isolate. All the other sequences of this study were retrieved from GenBank. All the sequences used to construct the phylogenetic tree are listed in Table 1; *Amaurodermacalcitum* D.H. Costa Rezende & E.R. Drechsler-Santos (FLOR:50931) (Costa-Rezende et al. 2017) was used as the outgroup taxon. Sequences were aligned with MAFFT online server (Katoh and Standley 2013), and manually adjusted using Bioedit v. 7.2.5 (Hall 1999). Alignments were checked and optimized manually when necessary. Maximum parsimony (MP) analysis was performed with PAUP v. 4.0b10 (Swofford 2002). Maximum likelihood analyses (ML) were executed on the CIPRES webportal (Miller et al. 2010), performed using RAxML-HPC2 on XSEDE v. 8.2.8 (Stamatakis 2014), and carried out using raxmlGUI v. 1.3.1 (Silvestro and Michalak 2011). The best fitting substitution model for each single gene partition and the concatenated data set were determined in MrModeltest 2.3 (Nylander 2004). Bayesian inference posterior probabilities (PP) with GTR+I+G model was used for each partition. Bayesian Markov Chain Monte Carlo (MCMC) analyses were conducted in MrBayes v. 3.2.2 (Huelsenbeck and Ronquist 2001). The number of generations was set at 1,000,000, with trees being sampled every 100 generations (a total of 10,000 trees), resulting in an average standard deviation of split frequencies below 0.01. Based on the tracer analysis, the first sampled topologies of 2000 trees representing 20% of burn-in phase were discarded. The remaining 8000 trees were used for calculating posterior probabilities (PP) in the majority rule consensus tree (Larget and Simon 1999).

**Table 1.** Fungal species and GenBank accession number of sequences used in this study.

Fungal species	Voucher	GenBank accession no.			References
		ITS	LSU	RPB2	
* Ganoderma applanatum *	Wei 5787a	KF495001	KF495011	–	GenBank
* G. applanatum *	SFC20141001–24	KY364255	–	KY393273	Jargalmaa et al. 2017
* G. australe *	HUEFS: DHCR 417	MF436676	MF436673	–	Costa–Rezende et al. 2017
* G. austroafricanum *	CMW 41454	KM507324	KM507325	–	Coetzee et al. 2015
* G. chalceum *	URM 80457	JX310812	JX310826	–	GenBank
* G. destructans *	CBS 139793	NR_132919	NG_058157	–	Coetzee et al. 2015
* G. destructans *	CMW 43670	KR183856	KR183860	–	Coetzee et al. 2015
* G. enigmaticum *	CBS 139792	NR_132918	NG_058156	–	Coetzee et al. 2015
* G. enigmaticum *	Ghana2/938397	KR014265	KR014266	–	GenBank
* G. gibbosum *	UB1	KU569556	KU570954	–	Bolaños et al. 2016
* G. gibbosum *	SPC2	KU569547	KU570946	–	Bolaños et al., 2016
* G. lingzhi *	Dai12441	JQ781869	–	–	Cao et al. 2012
* G. lingzhi *	Li245	JQ781863	–	–	Cao et al. 2012
* G. lingzhi *	Wu 1006–3	JQ781858	–	–	Cao et al. 2012
* G. lucidum *	K175217	KJ143911	–	–	Zhou et al. 2015
* G. lucidum *	Dai11593	JQ781852	–	–	Cao et al. 2012
* G. lucidum *	Dai2272	JQ781851	–	–	Zhou et al., 2015
* G. lucidum *	Rivoire 4195	KJ143909	–	–	Zhou et al. 2015
* G. multiplicatum *	CWN 04670	KJ143913	KJ143972	KJ143972	Zhou et al. 2015
* G. multiplicatum *	HMAS 242384	JF915409	–	JF915432	Wang et al. 2012
* G. multiplicatum *	Dai 9447	KJ143914	–	KJ143973	Zhou et al. 2015
* G. orbiforme *	URM 83332	JX310813	JX310827	–	Lima Júnior et al. 2014
* G. orbiforme *	URM 83334	JX310814	JX310828	–	Lima Júnior et al. 2014
* G. orbiforme *	URM 83335	JX310815	JX310829	–	Lima Júnior et al., 2014
* G. parvulum *	URM 83339	JX310817	JX310831	–	Lima Júnior et al. 2014
* G. parvulum *	URM 83340	JX310818	JX310832	–	Lima Júnior et al. 2014
* G. pfeifferi *	120818	AY884185	–	–	GenBank
* G. pfeifferi *	JV 0511/11	KF605660	–	–	GenBank
* G. resinaceum *	URM 83400	JX310824	JX310838	–	Lima Júnior et al. 2014
* G. resinaceum *	HSBU 200830	KT343303	–	–	GenBank
* G. resinaceum *	HMAS 86599	AY884177	–	JF915435	GenBank
* G. sessile *	UMNMN8	MG654281	–	–	GenBank
* G. sichuanense *	MFU16–2670	KY404119	–	–	Thawthong et al. 2017
* G. sichuanense *	HMAS 251145	JF915400	–	–	Wang et al. 2012
* G. sichuanense *	MFU16–2667	KY244061	–	–	Thawthong et al. 2017
* G. sichuanense *	MFU16–2668	KY244062	–	–	Thawthong et al. 2017
* G. tropicum *	HKAS: 76644	KC222317	–	–	Yang and Feng 2013
* G. tropicum *	Dai9724	JQ781879	–	–	Cao et al. 2012
* G. tropicum *	HMAS 263143	JF915410	–	–	Wang et al. 2012
*G.tropicu*m	Wu 0407–2	EU021458	–	–	Wang et al. 2009
* G. tropicum *	BCRC 37122	EU021457	–	–	Wang et al. 2009
* G. tropicum *	KUMCC 18–0046	**MH823539**	**MH823540**	**MH883621**	This study
* G. valesiacum *	CBS 428.84	JQ520218	–	–	Park et al. 2012
* Amauroderma calcitum *	FLOR: 50931	KR816528	KU315207	–	Costa–Rezende et al. 2017

**Figure 1.**
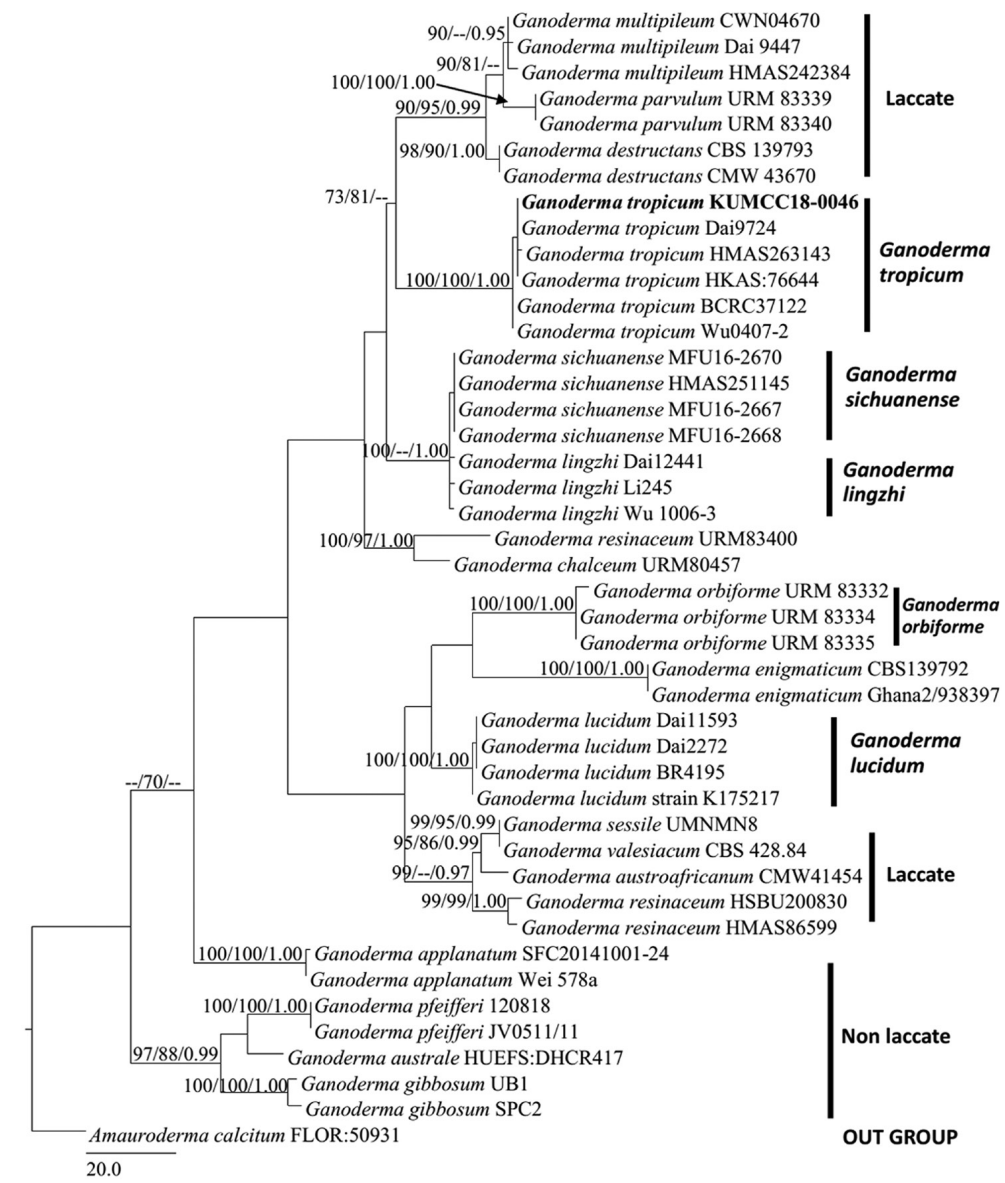
Phylogenetic tree (RAxML) obtained from the DNA sequence data of LSU, ITS, and RPB2 datasets. Bootstrap values (BS) from maximum likelihood (ML, left), Maximum parsimony (MP, middle) greater than 70% and Bayesian posterior probabilities (PP) greater than 0.95 are indicated above the nodes as MLBS/MPBS/PP. The tree is rooted with *Amaurodermacalcitum* FLOR:50931. Newly recorded species are indicated in black bold.

Phylogenetic trees and data files were figured in FigTree v. 1.4.0 (Rambaut 2012) and edited using Microsoft Office PowerPoint 2010 and exported to Adobe Illustrator CS v. 3. Maximum likelihood (ML) and Maximum parsimony (MP) bootstrap values, equal to or greater than 70%, and Bayesian Posterior Probabilities (BP) equal to or greater than 0.95, are presented above each node (Fig. 1).

### Optimal conditions for mycelial growth

Seven different solid culture media were evaluated to determine the optimal media for the mycelial growth of the *G.tropicum* strain KUMCC18-0046, namely Czapek’s agar (CZA), malt extract agar (MEA), potato dextrose agar (PDA), rose Bengal agar (RBA), yeast extract agar (YEA), yeast malt extract agar (YMA), and yeast extract peptone dextrose agar (YPD). The media formulae used are shown in Table 2. All media petri dishes were incubated at 25 °C under dark conditions. In order to discover the mycelial growth rate, colony diameter (mm) was measured; and colony averages calculated by averaging the vertical and horizontal lengths. Mycelial characteristics on the agar surfaces were recorded. Mycelial density was determined by following Kadiri (1998) as very scanty (+), scanty (2+), moderate (3+), somewhat abundant (4+), and abundant (5+). The optimal conditions, growth rate, and mycelial density were carried out in five replicates.

**Table 2.** Composition of culture media used in this study.

Agar media reagents	Agar media composition (g/L)						
	CZA	MEA	PDA	RBA	YEA	YMA	YPD
Potato infusion			4				
Malt extract		20				3	
Yeast extract					3	3	10
Peptone		6			5	5	20
Dextrose		20	20				20
Glucose						10	
Saccharose	30						
Sodium nitrate	33						
Di-potassium phosphate	1						
Magnesium sulfate	0.5			0.5			
Potassium chloride	0.5						
Ferrous sulfate	0.01						
Potassium dihydrogen phosphate				1			
Rose bengal				0.033			
Chloramphenicol				0.1			
Agar	15	15	15		15	20	15

The optimal media shown for mycelial growth was then used to determine the optimal pH for mycelial growth. pH was adjusted to 4, 5, 6, 7, 8, and 9 with 1N HCl and 1N NaOH. The optimal temperature for mycelial growth was determined by using the highest growth rates of media and pH conditions under different dark conditions; including 15 °C, 20 °C, 25 °C, 28 °C, 30 °C, and 35 °C. After 10 days of incubation, five replicates of colony diameter were measured and calculated. The colony diameter was measured as described above.

Data analysis was carried out using statistical programs (SPSS) with five replicates (*n* = 5). All data were compared to obtain a mean separation using Tukey’s test (*p* < 0.05) followed by post-hoc tests. The results are expressed in a one-way analysis of variance (ANOVA) analysis using the SPSS program (Softonic International SA, Barcelona, Spain).

## Results

### Phylogenetic analyses

Phylogenetic analyses were inferred from the combined LSU, ITS, and RPB2 sequences, comprising 44 taxa, including 19 *Ganoderma* species with *Amaurodermacalcitum* FLOR: 50931 as the outgroup taxon. The dataset comprised 2223 total characters, of which 1961 were constant, 176 variable characters were parsimony-informative, and 86 characters were parsimony-uninformative. The tree topologies were grouped into nine distinct clades, including five laccate clades of *G.tropicum*, *G.sichuanense*, *G.lingzhi*, *G.orbiforme*, *G.lucidum*, and two other laccate clades with one non-laccate clade, and an outgroup clade. The phylogenetic analyses showed considerably high support for the *G.tropicum* strain KUMCC18-0046 being closely related to the laccate *G.tropicum* isolates of China and Taiwan (MLBS = 100%/ MPBS = 100%/ PP = 1.00).

## Taxonomy

### Ganoderma tropicum

Taxon classificationFungiPolyporalesGanodermataceae

(Jungh.) Bres., Annales Mycologici 8(6): 586 (1910)

FOF number: FoF 05068

Fig. 2

#### Description based on specimen from Thailand.

**Basidiome.** Sessile, dimidiate. ***Pileus shape.*** Semicircular to dimidiate or conks, up to 7–12 cm in length and 4–8 cm in width, up to 1.5 cm thick. ***Pileus surface.*** Dark brown (9F5) at the base, slightly brownish red (10C8) at center, reddish gray (10B8) extending to the margin, light yellow (1A5) to yellow (2A6) under basidiocarp with grayish yellow (4C7) to brown (6D7 to 6F6) close to tube layer on upper surface of pileus glabrous, weakly to strongly laccate, glossy and shiny, smooth, spathulate, shallow sulcate several layers thick, consistency furrows, thicker at the base than the margin, covered by a thin and hard crust, and light in weight when dried. Context trimitic, irregular cuticle cells, mostly light yellow (1A5) to yellow (2A6) close to crust, grayish yellow (4C7), brown (6D7 to 6F6) to dark brown (9F5), near the tubes, dense context layer, thick near the base, tough to break when dried. ***Hymenophore.*** Grayish yellow (4C7). ***Basidiospores.*** Mostly oblong ellipsoid and broadly ellipsoid with double wall (ganodermoid) at maturity, (7.3–)7.6–10.1(10.8) × (10.1)10.6–13.3(13.9) μm (*x̄* = 9.1 × 12.2 μm, *n* = 50) (including myxosporium), (5.4–)5.6–8.3(8.6) × (8.3)8.4–12.5(12.9) μm (*x̄* = 7.1 × 10.6 μm, *n* = 50) μm (excluding outer myxosporium), light brown (6D6–6D8), reddish brown (9F6) to dark brown (9F8), usually with one end tapering, and usually overlaid by a hyaline myxosporium. ***Tubes.*** 2–7 mm long, up to 80–170 µm wide, and sulcate at different levels. ***Stipe.*** Lateral, up to 1.5 cm thick, dark brown (9F5). ***Margin.*** reddish gray (10B8), up to 0.3–0.7 cm thick, round, tough and hard, thicker towards the margin. ***Pore.*** Angular, 4–7 per mm; pore diameter up to 65–120 µm. ***Pore surface*** Pale yellow (2A3) to light yellow (2A5) and brown (6D7) to dark brown (6F6) when touched. ***Hyphal system*** Generative hyphae up to 0.80–2.85 μm (*x̄* = 1.45, *n* = 50) in diameter, colorless, thin-walled, some thick-walled, branched, with clamp connections; binding hyphae 1.00–3.10 µm (*x̄* = 2.05, *n* = 50), colorless, thin-walled, much-branched, clamped; skeletal hyphae up to 1.45–4.25 μm (*x̄* = 2.35, *n* = 50), colorless, thick-walled, unbranched or with a few branches in the distal end. ***Culture characteristics.*** Initially white (4A1) to yellowish white (4A2), pale yellow (4A3) when growing, become orange white (5A2), pale orange (5A3), light orange (5A4–6A5) and some reddish yellow (4A6) to dark brown (9F8) around the plugged circle of active mycelium after incubation for 3 weeks. ***Odor.*** Distinctive odor when fresh and dried.

*Ganodermatropicum* is diagnosed as having a distinctly dimidiate, smooth, spathulate pileus, with a laccate or glabrous dark brown slightly brownish red upper surface, usually tough when dried; margin usually has a reddish gray surface, round and hard; pore surface pale yellow when young, light yellow when mature, and becoming brown or dark brown when bruised; basidiospores are described as ellipsoid, with size range of (7.3–)7.6–10.1(10.8) × (10.1)10.6–13.3(13.9) μm (including myxosporium), (5.4–)5.6–8.3(8.6) × (8.3)8.4–12.5(12.9) μm (excluding outer myxosporium); context trimitic, abundant generative hyphae with branches; thin-walled, binding hyphae; and skeletal hyphae with clamp connections.

**Figure 2.**
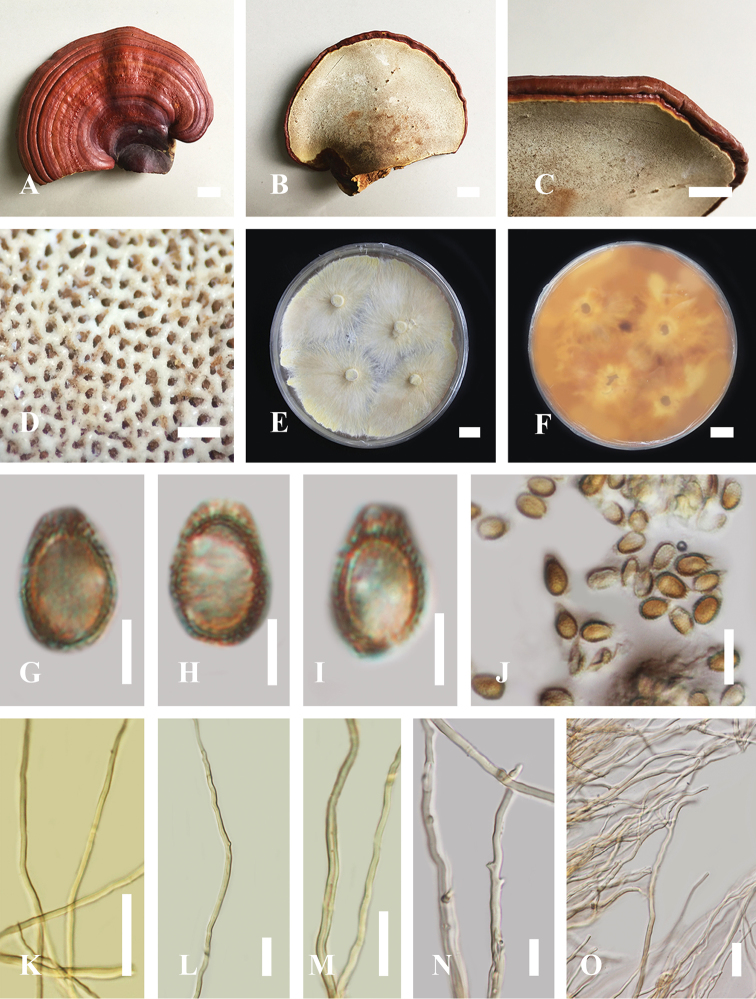
Morphology of *Ganodermatropicum* strain KUMCC18-0046 **A, B** Mature basidiocarps **C** Margin **D** Pore characteristics **E, F** Culture after incubation at 25 °C for 21 days **G, J** Basidiospore in KOH **K, L** Generative hyphae of context in KOH **M** Skeletal hyphae **N** Skeletal hyphae and binding hyphae **O** Sparing branch hyphae. Scale bars: 1 cm (**A–C**); 500 µm (**D**); 1 cm (**E, F**); 5 µm (**G–I**); 15 µm (**J**); 20 µm (**K, L, O**); 10 µm (**M, N**).

#### Habitat.

Solitary on living *Dipterocarpus* species in deciduous forests.

#### Specimen examined.

Thailand, Chiang Rai Province, 19°48'24"N, 100°03'54"E, 836 m, October, 2017.

##### Optimal media conditions for mycelial growth and characteristics of mycelial cultures

In our study of the seven different agar media, mycelial growth (mm), growth rates (mm/day), and mycelial density were screened as an indication of favorable growth of Thai *G.tropicum* (Table 3). After 10 days of incubation, the agar surface was fully colonized with a white (6A1) to pale orange (6A2–6A3) mycelium. The best mycelium colony diameter was observed on PDA, MEA, and YPD media, following YMA, RBA, YEA, and CZA, respectively.

**Table 3.** Effect of various agar media on mycelial growth (mm) and mycelial growth rates (mm/day) of *Ganodermatropicum* strain KUMCC18-0046, incubated at 25 °C for 10 days. Values with the same letter are not significantly different (*p* < 0.05).

**Agar media**	**Colony diameter**	**Growth rate**	**Mycelial density**
CZA	16.70 ± 0.13^e^	3.50	+
MEA	41.20 ± 0.12^a^	8.40	5+
PDA	42.20 ± 0.44^a^	8.50	4+
RBA	27.70 ± 0.08^c^	4.20	3+
YEA	21.00 ± 0.08^d^	5.70	2+
YMA	36.90 ± 0.13^b^	8.10	5+
YPD	40.40 ± 0.40^a^	8.40	5+

**Figure 3.**
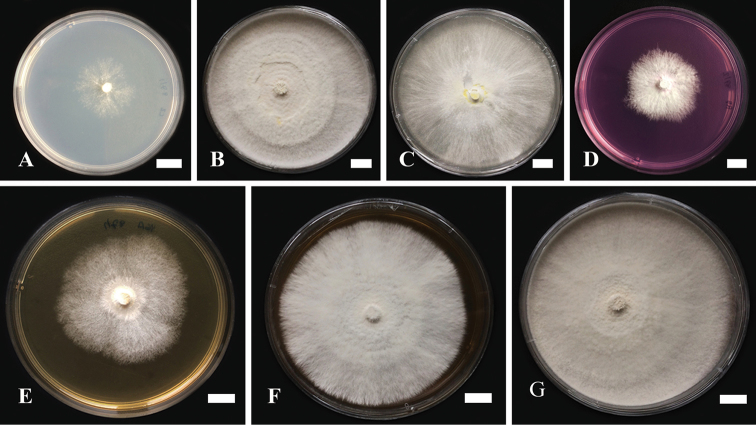
Characteristics of *Ganodermatropicum* strain KUMCC18-0046 mycelial cultures were incubated at 25 °C for 10 days on different agar media **A** Czapek’s agar (CZA) **B** Malt extract agar (MEA) **C** Potato dextrose agar (PDA) **D** Rose Bengal agar (RBA) **E** Yeast extract agar (YEA) **F** Yeast malt extract agar (YMA) **G** Yeast extract peptone dextrose agar (YPD). Scale bars: 1 cm.

Mycelial morphology and colony color characteristics differed on each agar media (Fig. 3). For instance, the morphological characteristics of *G.tropicum* growth on CZA medium were expressed as a very scanty, cotton colony (Fig. 3A). The colony on YEA medium was similar to that of CZA, although YEA exhibited greater density and biomass (Fig. 3E). Mycelial morphological characteristics on MEA and YPD were similar; both were expressed as an abundant (5+) massive cottony colony with orange white to pale orange (6A1–6A3) colony (Fig. 3B, G). The PDA medium, by contrast, showed a somewhat abundant (4+) white cotton colony (Fig. 3C), which was a slightly dark golden yellow (5A7) colony after 18 days of incubation (Fig. 2E, F). Moderate colony growth (3+) was observed on RBA (Fig. 3D). Abundant white massive cottony mycelia, with a radius from the center towards the edge of the petri dish, were observed on the YMA medium (Fig. 3F). Furthermore, filamentous colonies were observed in all media, except for CZA and YEA.

##### Optimal pH conditions for mycelial growth

All pH values from 4–9 were suitable for promoting mycelium growth of *G.tropicum*, however, the most favorable pH range was shown to be pH 7–8, followed by pH 9 (Table 4).

**Table 4.** Effect of pH on mycelial growth (mm) and mycelial growth rates (mm/day) of *Ganodermatropicum* strain KUMCC18-0046, incubated for 10 days. Values with the same letter are not significantly different (*p* < 0.05).

**pH**	**Colony diameter**	**Growth rate**	**Mycelial density**
4	30.50 ± 0.14^d^	5.50	3+
5	45.90 ± 0.10^c^	7.30	4+
6	46.80 ± 0.10^c^	8.10	4+
7	57.50 ± 0.12^ab^	8.50	5+
8	58.90 ± 0.05^a^	8.50	5+
9	56.10 ± 0.07^b^	8.10	5+

##### Optimal temperature conditions for mycelial growth

*Ganodermatropicum* mycelial growth increased when going up from 15–25 °C and 28 °C, after which it started to decline again, with the most suitable temperature for mycelial growth being between 25 °C and 28 °C. Although the mycelia could grow between 15–35 °C, growth appeared to be drastically suppressed at 15 °C and 35 °C (Table 5).

**Table 5.** Effect of temperature on mycelial growth (mm) and mycelial growth rates (mm/day) of *Ganodermatropicum* strain KUMCC18-0046, incubated for 10 days. Values with the same letter are not significantly different (*p* < 0.05).

Temperature (°C)	Colony diameter	Growth rate	Mycelial density
15	17.40 ± 0.07^c^	4.00	1+
20	29.70 ± 0.04^b^	6.30	3+
25	43.50 ± 0.06^a^	8.50	5+
28	43.70 ± 0.04^a^	8.50	5+
30	30.40 ± 0.13^b^	6.40	4+
35	17.90 ± 0.11^c^	4.30	2+

## Discussion

In this study, we introduce a new record of *G.tropicum* strain KUMCC18-0046, which was collected from Chiang Rai Province, Thailand. *Ganodermatropicum* serves as the sister group to *G.multipileum*, *G.parvulum*, and *G.destructans* (ML = 73%, MP = 81%). This finding is consistent with those of Wang et al. (2012), as well as Yang and Feng (2013), whose studies indicated that *G.tropicum* forms a sister clade with *G.multipileum*, exhibiting macro-morphological characteristics of strongly echinulate basidiospores (Wang et al. 2009). *Ganodermatropicum* and *G.multipileum* were also shown to be the sister groups of *G.lingzhi*, which is in the same clade of species distributed in China (Wang et al. 2012).

*Ganodermatropicum* has been widely reported in tropical areas; however, no specimens have been recorded in Thailand prior to this study (Wang et al. 2009; Zhang et al. 2015). The morphological characteristics of the *G.tropicum* strain from our study are similar to other *G.tropicum* specimens described from other tropical areas, including in mainland China (Cao et al. 2012; Wang et al. 2012; Hapuarachchi et al. 2018a), South America (Gottlieb and Wright 1999), and Taiwan (Wang et al. 2009). Although there is a high degree of variability in the macro-morphological characteristics of *Ganodermatropicum* specimens found around the world, certain common characteristics can be seen. These common characteristics include a distinct reddish-brown pileal surface, with sessile to dimidiate basidiocarps. Furthermore, there are some similarities between *G.tropicum* and other *Ganoderma* species. According to Cao et al. (2012), among the Chinese *Ganoderma* species, *G.flexipes*, *G.multipileum*, *G.sichuanense*, *G.lingzhi*, and *G.tsugae* are morphologically similar to *G.tropicum*, having a reddish-brown pileus surface, ellipsoid basidiospores, and cuticle cells.

However, *G.flexipesis* can be differentiated from *G.tropicum* by its small basidiocarps and long stipe, while *G.lingzhi* has usually distinctive sessile basidiocarps, a dark brown context, and mostly irregular cuticle cells (Cao et al. 2012; Wang et al. 2012). *Ganodermamultipileum* is distinguished from *G.tropicum* by having mostly concentric growth zones in context, and varying the homogeneous context when maturity (Wang et al. 2012). *Ganodermasichuanense* is separated from *G.tropicum* by its usually formed flabellate to reniform, concave or convex basidiocarps, and also by its ovoid basidiospores which are truncate at the apexes (Yao et al. 2013). *Ganodermatsugae* is separated from *G.tropicum* by absence of the melanoid bands, and also thin dissepiments when mature (Wang et al. 2012).

Our morphological analyses show that the Thai *G.tropicum* strain has a semicircular to dimidiate pileus, a pileus size between 4–8 cm in width, 7–12 cm in length, and up to 1.5 cm thick. The basidiospores are mostly oblong ellipsoid and broadly ellipsoid in shape, with double walls, (7.3–)7.6–*8.2*–10.1(–10.8) × (10.1–)10.6–*11.7*–13.3(–13.9) μm (*x̄* = 9.1 × 12.2 μm, *n* = 50), and (5.4–)5.6–*7.1*–8.3(–8.6) × (8.3)8.4–*10.8*–12.5(–12.9) μm (*x̄* = 7.1 × 10.6 μm, *n* = 50) mm (excluding outer myxosporium); the pore surface is pale yellow (2A3) with pore are 4–7 per mm, and the tubes are 2–7 mm long with a light yellow to dark brown context. The original description of *G.tropicum* has the basidiospores fasciculate, 7–9 × 10–12 µm with 4–5 µm of hymenia hyphae (Tai et al. 1979). These characteristics are in accordance with the basidiospore sizes we recorded for the Thai strain of *G.tropicum*. The strain of *G.tropicum* from South America shares much in common with the Thai strain; however, notable differences in the South American strain include light brown ovoid basidiospores, a pileus of dark to black coloring at the base, and a blunt to slightly round margin. Our results of *G.tropicum* are in accordance with the description of Hapuarachchi et al. (2018a), who described specimens of a Chinese strain of *G.tropicum* collected from Hainan Province. This Hainan strain had the following characteristics: pileus size 4–8 × 2.5–6 cm, up to 1 cm thick at the base, basidiospores were described as (10.8–)11.2–*12.1*–12.8(–13.1) × (8.3–)9.6–*10.1*–10.8(11.1) μm (with myxosporium) and (7.9–)8.8–*9.1*–10.2(–10.8) × (5.8–)6.4–*7.3*–7.8(–9.8) μm (without myxosporium), with shared white to orange pore surface.

The optimal conditions for mycelial growth were investigated based on medium, pH, and temperature. The best growth rates were obtained using PDA, MEA, and YPD media. These three media are composed of high concentrations of dextrose as a carbon source, while various forms of carbon sources have been reported as affecting fungal mycelial growth (Simonic et al. 2008; Dang et al. 2018). Although identical fungal species are able to grow on different agar media, the morphological characteristics of the mycelia can be expressed differently, and we therefore conclude that each ingredient in each agar medium affects the morphological characteristics of the resultant culture. The optimal pH was evaluated by using the PDA media, and pH 7–8 was found to be the optimal pH range. Here, we found that *G.tropicum* grows well in an alkali pH range, as its fruiting body was also collected on the substrate at pH 8 in nature (data not shown). The optimal PDA media at pH 7, incubated within the temperature range at 25–28 °C, were found to be the most suitable for *G.tropicum* mycelial growth, while temperatures lower than 15 °C or higher than 30 °C are not suitable for mycelial growth. Luangharn et al. (2017) reported that the non-laccate Thai strain of *G.australe* grew well on PDA media, at a pH of 7–8 and at temperature range of 25–30 °C. This study revealed similar mycelial growth conditions with other *Ganoderma* species that have been previously evaluated (Lee et al. 2008; Jo et al. 2009; Magday et al. 2014). In light of medicinal mushroom consumption trends, *G.tropicum* has a high potential for commercial production. Further studies will evaluate the best method to optimize the mushroom spawn and growing substrate for bringing Thai *G.tropicum* cultivation into high-yield production, and also establish whether other *Ganoderma* species remain to be discovered in Thailand.

## Conclusion

This study confirmed the new record of *Ganodermatropicum* from Northern Thailand based on morphological characteristics together with phylogenetic analyses. The optimal conditions for promoting the mycelial growth of *G.tropicum* were investigated and the best media and pH for mycelia growth were found to be PDA, MEA, and YPD media at pH 7–8, respectively. The optimal temperature was found to be 25–30 °C.

## Supplementary Material

XML Treatment for Ganoderma tropicum
